# Factors Associated With a Higher Score of Burnout in a Population of 860 French Psychiatrists

**DOI:** 10.3389/fpsyt.2020.00371

**Published:** 2020-05-07

**Authors:** Philippe Nuss, Cedric Tessier, Marc Masson, Philippe Fossati, Raphaël Gaillard, Nathanaël Lapidus, David Gourion

**Affiliations:** ^1^AP-HP, Service de Psychiatrie et de Psychologie Médicale, Paris, France; ^2^Sorbonne Université, INSERM, Centre de Recherche Saint-Antoine, CRSA, UMRS 938, Paris, France; ^3^Nightingale Hospitals, Château de Garches Clinic, Garches, France; ^4^Department of Psychiatry, Sainte-Anne Hospital, Paris, France; ^5^Institut du cerveau et de la moelle epiniere, INSERM U 1127, Paris, France; ^6^Sorbonne Université, CNRS UMR 7225, Paris, France; ^7^AP-HP, Department of Adult Psychiatry, Pitie Salpetriere Hospital, Paris, France; ^8^Centre Hospitalier Sainte Anne, Service Hospitalo-universitaire de Psychiatrie, Paris, France; ^9^Sorbonne Université, INSERM, Institut Pierre Louis d’Epidémiologie et de Santé Publique IPLESP, AP-HP.Sorbonne Université, Public Health Department, Saint-Antoine Hospital, Paris, France; ^10^Hautes etudes de commerce (HEC) Paris Business School, Jouy-en-Josas, France

**Keywords:** burnout - professional, psychology, psychiatrist, public - private, risk factors, stressful life events

## Abstract

Burnout rates are estimated to be twice as high among healthcare professionals as in the general working population, and studies indicate rising incidence. The present study aimed to identify the contextual factors associated with self-reported burnout rates among French psychiatrists. A total of 860 French or French-speaking psychiatrists completed an online questionnaire when they registered for a major psychiatric conference. The Copenhagen Burnout Inventory, a validated scale that independently appraises personal, work- and patient-related dimensions, was used to assess the degree of perceived burnout. Respondents were divided into lower risk and higher risk groups. The latter contained the 25% of individuals who scored the highest on each of the three dimensions of the CBI scale. Univariate analysis showed that private practice was associated with lower levels of risk on the personal and work-related dimensions. Working for the public sector and long hours were both associated with a higher score on the work-related dimension. Interestingly, none of the variables we investigated, except from poor atmosphere at work, correlated with the patient-related dimension. Among public-sector psychiatrists, female gender, longer hours, and more consultations per week were associated with a higher score on the work-related dimension. Working four or more night shifts per month was significantly associated with a higher score of burnout risk on all three dimensions. Private- and public-sector practitioners who mainly treated patients with schizophrenia had a higher score of burnout risk. Multivariate analysis showed that a poor atmosphere at work, longer hours, and working four or more night shifts were significantly associated with higher score of burnout risk. A nonreassuring working environment and more stressors while treating patients each had a possibly negative impact. Although this study only examined the factors that distinguish between clinicians with the lowest *versus* highest CBI burnout risk scores, it opens up important avenues for research and development of programs to reduce burnout risk within the French healthcare system.

## Introduction

Burnout is classically observed in the occupational context and is most often described among human service workers. Burnout is characterized either causally, as “a state of physical, emotional and mental exhaustion that results from long-term involvement in work situations that are emotionally demanding” (1), or conceptually, as “weariness or exasperation brought about by the individual’s dedication to a cause or way of life that failed to meet their expectations” (2). Taxonomically speaking, burnout refers to stress (3), but this occupational phenomenon is not classified as a medical condition. The commonly used triad describing burnout as emotional exhaustion, depersonalization, and low sense of personal accomplishment related to one’s work (4) was proposed in the wake of the original formulation of *occupational burnout* by Herbert J. Freudenberger (5). The conceptualization of burnout was recently refined in the ICD-11 classification (3). Here, burnout refers to a pathological condition related to significant and prolonged stress in the workplace attributed to work overload, inadequate resources to meet the demands of work, limited control over one’s work schedule and lack of autonomy, and inadequate support from colleagues, supervisors, and coworkers. The core criteria of burnout include feelings of energy depletion or exhaustion, increased mental distance from one’s job (or feelings of negativity or cynicism related to one’s job), and reduced professional efficacy. The potential overlap between burnout and depression is still subject to debate (6–9), despite a large body of research pointing to a continuum between these two constructs (10). Because of the lack of categorical criteria for burnout, studies compare scores yielded by dimensional psychopathological scales of both burnout and depression but without the ability to use categorical cut-offs to decide whether these entities are discrete or continuous. In addition, depressogenic and burnout factors can coexist within a given working environment and variously affect individuals, blurring the psychopathological picture.

Psychiatrists are vulnerable to experiencing burnout, partly for reasons common to all healthcare professionals, partly for reasons specific to their activity. First, psychiatrists are both the instigators and the tools of their patients’ treatment. An incompressible amount of personal emotional involvement and shared values is intimately bound up with a technical determination to achieve treatment effectiveness (11). Working while still in their residency with marginalized populations or in a context of suicide and violence—a frequent situation in public-sector practice—is liable to increase practitioners’ risk of burnout (11). Another predisposing and precipitating factor in psychiatrists is the frequency of role conflict conditions in the work setting (12). While psychiatrists are trained to appraise symptoms, foresee changes along a lifelong trajectory, and use a verbal approach, they are often required to deliver short-term and mainly biological treatment responses (13). In addition, psychiatrists may have predisposing personality traits such as neuroticism, compared with physicians in other disciplines (14), and may be more prone to internalize their stressful experiences (15). Factors external to the doctor–patient therapeutic relationship, such as working conditions, may be yet more potent stressors for psychiatrists, just as they are detrimental for all physicians. These factors can contribute to the risk either directly, by inducing stress, or indirectly, by reducing psychiatrists’ ability to build the therapeutic alliance required for treatment to be effective. The rapidly changing modes of service delivery, combined with limited resources, conflicting and paradoxical injunctions from government and healthcare/corporate management, and an ever heavier clerical burden are highly distressing to practitioners as they undermine the core conditions for care (16–18). Organizational factors, such as negative leadership behaviors and time-consuming bureaucratic issues, along with the general public’s ambivalence toward psychiatry and psychiatrists, further contribute to occupational burnout (16, 17, 19, 20). Moreover, the increasing complexity and rapidity of change in the understanding of mental disorders, in terms of classification, neurobiology, psychopharmacology, and desirable outcome, contrast with the oversimplified and rapidly changing objectives and assessment tools adopted by institutions to measure clinical performance (13). Interestingly, despite these hurdles, medical doctors continue to express a strong desire to provide good-quality care, regardless of circumstances, and display engagement characterized by vigor, dedication, and absorption in work, which may well heighten the risk of burnout risk (21).

There is a striking absence of consensus on an operational definition of burnout, and this was highlighted in a recently published meta-analysis of 182 studies on burnout involving 109,628 individuals in 45 countries published between 1991 and 2018 (22). At least 142 unique definitions were found to describe overall burnout or burnout subscale criteria. Political, managerial, and ultimately financial hurdles, along with genuine scientific issues, may explain this flagrant lack of an agreed definition despite a growing burnout epidemic.

For the present study, we chose the Copenhagen Burnout Inventory (CBI) (23) over the Maslach Burnout Inventory (MBI) (4), which is mostly based on the notion of depersonalization, and is more quantitative than qualitative. Symptoms in psychiatry are mostly put into perspective and considered in a web of interactions and meanings, rather than being considered in isolation. This requires clinicians to combine a subjective style in their relations with patients and a rigorously analytic approach in order to gain an overall picture. This is why the MBI, which refers to work independently of how it is practiced, seems less suited to psychiatrists. By contrast, the CBI includes a patient-related subcomponent and appraises the perceived distance between clinicians’ expectations and their experienced reality and expectations, and this is therefore the tool we chose. Alongside the patient-related dimension, the CBI independently measures the personal and work-related dimensions that can shed light on the stressful effects of personality traits and working conditions. Our aim was to examine the factors that differentiate between clinicians with lower *versus* higher CBI burnout risk scores in each of the patient-, personal-, and work-related dimensions.

## Participants and Methods

### Participants

The sample comprised French or French-speaking psychiatrists from both the public and private sectors. An invitation to fill in an online questionnaire designed to appraise the risk of burnout was displayed on the registration page of Encéphale, a major psychiatric conference held in France each year. There were no specific incentives for filling in this questionnaire. This study was exempt from ethics approval as the French Ethics law on experimentation on humans (Loi Jardé, 12–17 June 2017, in accordance with the Declaration of Helsinki) clearly states that the evaluation of the professional practices is not under its supervision. This information has been confirmed after solicitation by the Saint-Antoine Ethical Committee on May 29 2019.

### Burnout Scale

The CBI scale is composed of three independent subscales measuring personal- (6 items), work- (7 items), and patient- (6 items) related dimensions. The responses for twelve items are graduated in frequencies along a five-point Likert scale ranging from ‘never/almost never’ (0) to ‘always’ (4). The seven remaining items are categorized in intensity ranging from ‘a very low degree’ (0) to ‘to a very high degree’ (4). CBI scores were calculated for each dimension by summing the scores on all the relevant items for each participant. Personal questions in the CBI include “How often do you feel emotionally exhausted?” and “How often do you feel weak and susceptible to illness?” Work-related burnout is appraised *via* questions such as “Is your work emotionally exhausting?” and “Do you feel that every working hour is tiring for you?”, while the patient-related dimension is evaluated with questions like “Do you find it hard to work with patients?” and “Do you sometimes wonder how long you will be able to continue working with patients?”

### Additional Questions

Participants were asked four additional contextual questions. For the first one, they had to evaluate their workplace climate on a 3-point scale: 1 (*Reassuring*), 2 (*Stressful*), and 3 (*Extremely stressful*). Responses to the remaining three questions were provided on a 3-point scale: 1 (*Not at all*), 2 (*A little*), and 3 (*A lot*). They explored (i) the extent to which new societal demands for care/new challenges were affecting their perceived stress, (ii) the impact that rapid advances in scientific knowledge or imposed guidelines were having on their practice, and (iii) whether they were confronted with stressful situations in the course of their clinical practice.

Participants were also given a space where they could express themselves anonymously and add comments about their perceived stress.

### Definitions of At-Risk Groups

In the first analysis, a total CBI score was obtained for each dimension by summing the scores on the relevant items for each participant. Three severity groups were initially identified: nonvulnerable (<50% of total CBI score), vulnerable (50-74% of total CBI score), and at-risk (≥75% of total CBI score). However, there were too few participants in the at-risk groups to conduct statistical comparisons, with just 12.1% for the personal-related score, 7.8% for the work-related score, and 2.3% for the patient-related score, and ultimately only 4% when the three CBI dimensions scores were summed.

In the second analysis, we sought to determine a cut-off score to identify a more suitable at-risk group in the context of the present survey. We found a compromise between identifying individuals with the highest total CBI score and forming a large enough sample to run comparisons in order to study the factors associated with a higher risk of burnout. Reasoning that a higher-risk population could be identified independently of the total CBI score, we defined it as the 25% of individuals who scored the highest on each of the three dimensions.

### Statistical Analysis

For each CBI subscale, responses of higher-risk (HR) and lower-risk (LR) groups were described as mean ± standard deviation for quantitative variables and counts (percentages) for categorical variables. Results were stratified according to responders’ practice (public *vs.* private). In a univariate analysis, responses were compared between HR and LR groups with the use of Mann–Whitney–Wilcoxon tests for quantitative variables and Fisher’s exact tests for categorical variables. A multivariate logistic regression model selection was carried out to identify variables independently associated with HR on the global CBI scale, using the Akaike information criterion (AIC). Logistic regression results are reported with odds ratios (OR) with their 95% confidence intervals (CI). For all tests, differences were considered significant at the 0.05 threshold. All analyses were performed using R Statistical Software version 3.5 (Foundation for Statistical Computing, Vienna, Austria).

## Results

### Participants

A total of 1,010 online questionnaires were filled in, of which 860 were submitted prior to the conference and included in this analysis. Women represented 59.1% of respondents, with a higher proportion among public-sector practitioners than among private-sector ones (63.9 *vs.* 47.9%). The participants’ mean age was 45.5 years (public: 42.9 ± 11.9 years; private: 51.4 ± 10.5 years, p < 0.001).

### CBI Scores

Cronbach’s alpha estimations [95% confidence interval] for personal, work-related, and patient-related subscales were 0.90 [0.89, 0.91], 0.90 [0.89, 0.91], and 0.89 [0.88, 0.90], respectively. We divided the sample into two groups: LR *versus* HR. The HR group contained the 25% of individuals who scored the highest on each of the three CBI dimensions. Results are set out for each dimension, first for the whole sample, then for public- *versus* private-sector practitioners (**Tables 1**–**3**). Interestingly, for the patient-related dimension, the HR and LR groups did not differ on associated variables (except for a poor atmosphere at work), indicating that the patient-doctor relationship barely contributed to severity in the HR group.

**Table 1:** Variables studied for the personal dimension of the Copenhagen Burnout Inventory Values and statistical significance for the comparison between lower (LR) and higher (HR) risk of burnout are provided for the whole sample and the public- and private-sector subsamples.

Personal Dimension	Total Population (N = 840)			Public sector (n = 587)			Private sector (n = 253)		
Burnout Risk	LR	HR	*p*	LR	HR	*p*	LR	HR	*p*
Variables									
**Female n (%)**	353 (56.5)	142 (66.0)	0.016	254 (61.2)	120 (69.8)	0.059	99 (47.1)	22 (51.2)	0.737
**Mean age in years (SD)**	46.1 (12.3)	43.6 (11.5)	0.016	43.4 (12.2)	42.0 (11.3)	0.399	51.6 (10.6)	50.2 (9.9)	0.446
**Sector n (%)**			<0.001			0.925			N/A
Private	210 (33.6)	43 (20.0)		0	0		210 (83.0)	43 (17.0)	
Public	260 (41.6)	109 (50.7)		260 (62.7)	109 (63.4)		0	0	
Mixed	155 (24.8)	63 (29.3)		155 (37.3)	63 (36.6)		0	0	
**Place of work n (%)**			<0.001			0.738			0.006
Private sector	166 (26.6)	28 (13.0)		4 (1.0)	1 (0.6)		162 (78.3)	27 (67.5)	
University hospital	91 (14.6)	37 (17.2)		91 (21.9)	36 (20.9)		0	0	
General hospital	50 (8.0)	23 (10.7)		46 (11.1)	23 (13.4)		4 (1.9)	0	
Psychiatric hospital	146 (23.4)	79 (36.7)		144 (34.7)	75 (43.6		2 (1)	4 (10)	
Consultation clinic	57 (9.1)	14 (6.5)		57 (13.7)	14 (8.1)		0	0	
Other	115 (18.4)	34 (15.8)		73 (17.6)	23 (13.4)		39 (18.8)	9 (22.5)	
**No. years after graduation n (%)**			0.011			0.257			0.337
Residency	37 (5.9)	18 (8.4)		37 (8.9)	18 (10.5)		0 (0)	0 (0)	
1–5	141 (22.6)	58 (27.0)		121 (29.2)	53 30.8)		20 (9.5)	5 (11.6)	
6–10	88 (14.1)	32 (14.9)		64 (15.4)	27 (15.7)		24 (11.4)	5 11.6)	
11–20	117 (18.7)	44 (20.5)		63 (15.2)	30 (17.4)		54 (25.7)	14 (32.6)	
More than 20	242 (38.7)	63 (29.3)		130 (31.3)	44 (25.6)		112 (53.3)	19 (44.2)	
**Atmosphere at work n (%)**			<0.001			<0.001			0.067
Very difficult	8 (1.3)	19 (8.8)		8 (1.9)	17 (9.9)		0 (0)	2 (4.7)	
Difficult	84 (13.4)	58 (27).0		73 (17.6)	51 (29.7)		11 (5.2)	7 (16.3)	
Good	352 (56.3)	104 (48.4)		270 (65.1)	93 (54.1)		82 (39.0)	11 (25.6)	
Excellent	109 (17.4)	19 (8.8)		59 (14.2)	10 (5.8)		50 (23.8)	9 (20.9)	
No answer	72 (11.5)	15 (7.0)		5 (1.2)	1 (0.6)		67 (31.9)	14 (32.6)	
**Mean no. hours worked per week (SD)**	43.1 (11.0)	45.5 (10.3)	<0.001	42.4 (10.5)	45.1 (9.6)	<0.001	44.4 (11.9)	47.4 (12.7)	0.110
**Mean no. consultations per week (SD)**	45.6 (33.1)	42.2 (29.5)	0.266	33.7 ((23.6)	36.1 (25.8)	0.412	69.1 (36.4)	66.8 (30.7)	0.904
**Mean no. night shifts per month (SD)**	–	–	N/A	1.94 (2.2)	2.35 (2.6)	0.146	–	–	N/A
**No. night shifts per month**			N/A			0.056	–	–	N/A
0–3 (SD)	–			351 (84.6)	134 (77.9)		–	–	
4 or more (SD)	–			64 (15.4)	38 (22.1)		–	–	
**Main disorder treated by category**			<0.001			0.004			<0.001
Addiction	28 (4.5)	10 (4.7)		25 (6.0)	7 (4.1)		3 (1.4)	3 (7.0)	
Autism	16 (2.6)	18 (8.4)		16 (3.9)	17 (9.9)		0 (0)	1 (2.3)	
Schizophrenia/psychosis	187 (29.9)	88 (40.9)		182 (43.9)	82 (47.7		5 (2.4)	6 (14.0)	
Anxiety disorders	72 (11.5)	7 (3.3)		27 (6.5)	4 (2.3		45 (21.4)	3 (7.0)	
Mood disorders	262 (41.9)	68 (31.6)		130 (31.3)	41 (23.8)		132 (62.9)	27 (62.8)	
Personality disorders	60 (9.6)	24 (11.2)		35 (8.4)	21 (12.2)		25 (11.9)	3 (7.0)	
**Head of department n (%)**	133 (21.3)	56 (26.0)	0.155	115 (27.7)	51 (29.7)	0.614	18 (8.6)	5 (11.9)	0.559
**Working alone n (%)**	159 (25.4)	47 (21.9)	0.313	43 (10.4)	21 (12.2)	0.561	116 (55.2)	26 (60.5	0.614

**Table 2:** Variables studied for the work-related dimension of the Copenhagen Burnout Inventory.

Work-Related Dimension	Total population (N = 840)			Public sector (n = 587)			Private sector (n = 252)		
Burnout Risk	LR	HR	*p*	LR	HR	*p*	LR	HR	*p*
Variables									
**Female n (%)**	333 (56.7)	157 (63.1)	0.091	239 (68.8)	130 (60.5)	0.054	94 (49.0)	27 (45.0)	0.658
**Mean age in years (SD)**	45.8 (12.4)	44.6 (11.4)	0.259	42.9 (12.1)	43.0 (11.5)	0.646	51.9 (10.7)	49.4 (9.6)	0.102
**Sector n (%)**			0.015			0.169			N/A
Private	192 (32.7)	60 (24.1)		0 (0.0)	0		192 (76.2)	60 (23.8)	
Public	241 (41.1)	127 (51.0)		241 (61.0)	127 (67.2)		0	0	
Mixed	154 (26.2)	62 (24.9)		154 (39.0)	62 (32.8)		0	0	
**Place of work n (%)**			0.013			0.134			0.429
Private sector	149 (25.4)	45 (18.1)		3 (0.8)	2 (1.1)		146 (76.0)	43 (71.7)	
University hospital	92 (15.7)	35 (14.1)		92 (23.3)	34 (18.0)		0 (0.0)	0 (0.0)	
General hospital	44 (7.5)	29 (11.6)		41 (10.4)	28 (14.8)		3 (1.6)	1 (1.7)	
Psychiatric hospital	141 (24.0)	81 (32.5)		137 (34.7)	79 (41.8)		4 (2.1)	2 (3.3)	
Consultation clinic	48 (8.2)	23 (9.2)		48 (12.2)	23 (12.2)		0 (0.0)	0 (0.0)	
Other	113 (19.2)	36 (14.5)		74 (18.8)	23 (12.2)		39 (20.4)	13 (21.7)	
**No. years after graduation (%)**			0.384			0.722			0.191
Residency	36 (6.1)	18 (7.2)		36 (9.1)	18 (9.5)		0 (0.0)	0 (0.0)	
1–5	139 (23.7)	60 (24.1)		119 (30.1)	35 (29.1)		20 (10.4)	5 (8.3)	
6–10	85 (14.5)	35 (14.1)		66 (16.7)	25 (13.2)		19 (9.9)	10 (16.7)	
11–20	106 18.1)	53 (21.3)		58 (14.7)	34 (18.2)		48 (25.0)	19 (31.7)	
More than 20	221 (37.6)	83 (33.3)		116 (29.4)	57 (30.2)		105 (54.7)	26 (43.3)	
**Atmosphere at work n (%)**			<0.001			<0.001			0.009
Very difficult	8 (1.4)	19 (7.6)		8 (2.0)	17 (9.0)		0 (0.0)	2 (3.3)	
Difficult	80 (13.6)	63 (25.3)		72 (18.2)	53 (28.0)		8 (4.2)	10 (16.7)	
Good	329 (56.0)	124 (49.8)		254 (64.3)	105 (55.6)		75 (39.1)	19 (31.7)	
Excellent	104 (17.7)	23 (9.2)		57 (14.4)	12 (6.3)		47 (24.5)	11 (18.3)	
No answer	66 (11.2)	20 (8.0)		4 (1.0)	2 (1.1)		62 (32.3)	18 (30.0)	
**Mean no. hours worked per week (SD)**	42.8 (11.0)	45.8 (10.3)	< 0.001	42.3 (10.5)	44.9 (9.7)	0.009	43.7 (12.1)	48.6 (11.9)	0.003
**Mean no. consultations per week (SD)**	44.1 (32.6)	45.8 (31.0)	0.270	32.8 (23.6)	37.5 (25.9)	0.035	67.3 (36.2)	71.9 (31.7)	0.163
**Mean no. night shifts per month (SD)**	–	–	N/A	1.96 (2.19)	2.32 (2.60)	0.317	–	–	NA
**No. night shifts per month**			N/A			0.080			NA
0–3 (SD)	–	–		334 (84.6)	148 (78.3)		–	–	
4 or more (SD)	–	–		61 (15.4)	41 (21.7)		–	–	
**Main disorder treated by category**			<0.001			<0.001			0.905
Addiction	27 (4.6)	10 (4.0)		23 (5.8)	8 (4.2)		4 (2.1)	2 (3.3)	
Autism	19 (3.2)	15 (6.0)		19 (44.8)	14 (7.4)		0 (0.0)	1 (1.7)	
Schizophrenia/psychosis	170 (29.0)	104 (41.8)		164 (41.5)	99 (52.4)		6 (3.1)	5 (8.3)	
Anxiety disorders	66 (11.2)	13 (5.2)		25 (6.3)	6 (3.2)		41 (21.4)	7 (11.7)	
Mood disorders	255 (43.4)	74 (29.7)		134 (33.9)	37 (19.6)		121 (63.0)	37 (61.7)	
Personality disorders	50 (8.5)	33 (13.3)		30 (7.6)	25 (13.2)		20 (10.4)	8 (13.3)	
**Head of department n (%)**	122 (20.8)	65 (26.1)	0.084	107 (27.1)	58 (30.7)	0.325	15 (7.8)	7 (11.7)	0.431
**Working alone n (%)**	147 (25.0)	59 (23.7)	0.726	39 (9.9)	25 (13.2)	0.257	108 (56.2)	34 (56.7)	1

**Table 3:** Variables studied for the patient-related dimension of the Copenhagen Burnout Inventory.

Patient-Related Dimension	Total population (N = 819)			Public sector (n = 601)			Private sector (n = 245)		
Burnout Risk	LR	HR	*p*	LR	HR	*p*	LR	HR	*p*
Variables									
**Female n (%)**	360 (60.0)	121 (55.3)	0.229	271 (64.7)	93 (60.0)	0.329	89 (49.2)	28 (43.8)	0.471
**Mean age in years (SD)**	45.4 (12.1)	45.3 (12.1)	0.880	43.0 (11.9)	42.5 (11.9)	0.587	50.9 (10.7)	52.0 (10.0)	0.506
**Sector n (%)**			0.937			0.846			NA
Private	181 (30.2)	64 (29.2)		0 (0)	0 (0)		181 (73.9)	64 (26.1)	
Public	263 (43.8)	99 (45.2)		263 (62.8)	99 (63.9)		0 (0)	0 (0)	
Mixed	156 (26.0)	56 (25.6)		156 (37.2)	56 (36.1)		0 (0)	0 (0)	
**Place of work n (%)**			0.282			0.344			0.636
Private sector	139 (23.2)	51 (23.3)		4 (1.0)	1 (0.6)		135 74.6)	50 (78.1)	
University hospital	98 (16.3)	26 (11.9)		98 (23.4)	26 (16.8)		0 (0)	0 (0)	
General hospital	51 (8.5)	20 (9.1)		48 (11.5)	20 (12.9)		3 (1.7)	0 (0)	
Psychiatric hospital	152 (25.3)	67 (30.6)		148 (35.3)	65 (41.9)		4 (2.2)	2 (3.1)	
Consultation clinic	47 (7.8)	22 (10.0)		47 (11.2)	62 (14.2)		0 (0)	0 (0)	
Other	113 (18.8)	33 (15.1)		74 (17.6)	21 (13.5)		39 (21.6)	12 (18.8	
**No. years after graduation n (%)**			0.973			0.555			0.407
Residency	40 (6.7)	14 (6.4)		40 (9.5)	14 (9.0)		0 (0)	0 (0)	
1–5	138 (23.0)	55 (25.1)		116 (27.7)	53 (34.2)		22 (12.2)	2 (3.1)	
6–10	95 (15.8)	25 (11.4)		72 (17.2)	19 (12.3)		23 (12.7)	6 (9.4)	
11–20	109 (18.2)	47 (21.5)		67 (16.0)	24 (15.5)		42 (23.2)	23 (35.9)	
More than 20	218 (36.8)	78 (35.6)		124 (29.6)	45 (29.0)		94 (51.9)	33 (51.6)	
**Atmosphere at work n (%)**			<0.001			<0.001			0.009
Very difficult	12 (2.0)	15 (6.8)		11 (2.6)	14 (9.0)		1 (0.6)	1 (1.6)	
Difficult	89 (14.8)	50 (22.8)		80 (19.1)	42 (27.1)		9 (5.0)	8 (12.5)	
Good	328 (54.7)	118 (53.9)		261 (62.3)	83 (60.0)		67 37.0)	25 (39.1)	
Excellent	109 (18.2)	14 (6.4)		62 (14.8)	5 (3.2)		47 (26.0)	9 (14.1)	
No answer	62 (10.3)	22 (10.0)		5 (1.2)	1 (0.6)		57 (31.5)	21 (32.8)	
**Mean no. hours worked per week (SD)**	43.7 (10.6)	44.4 (11.0)	0.299	43.3 (9.9)	43.3 (10.5)	0.809	44.5 (11.9)	47.1 (11.7)	0.154
**Mean no. consultations per week (SD)**	44.9 (29.9)	46.6 (32.7)	0.213	33.8 (23.9)	36.8 (25.7)	0.233	68.0 (36.0)	70.4 (32.5)	0.517
**Mean no. night shifts per month (SD)**	–	–	N/A	1.96 (2.23)	2.35 (2.52)	0.080	–	–	NA
**No. night shifts per month**			N/A			0.106			NA
0–3 (SD)	–	–		353 (84.2)	121 (78.1)		–	–	
4 or more (SD)	–	–		66 (15.8)	34 (21.9)		–	–	
**Main disorder treated by category**			0.254			0.271			0.388
Addiction	27 (4.5)	10 (4.6)		24 (5.7)	7 (4.5)		3 (1.7)	3 (4.7)	
Autism	21 (3.5)	13 (5.9)		21 (5.0)	12 (7.1)		0 (0)	1 (1.6)	
Schizophrenia/psychosis	185 (30.8)	81 (37.0)		177 (42.2)	78 (50.3)		8 (4.4)	3 (4.7)	
Anxiety disorders	61 (10.2)	16 (7.3)		26 (6.2)	5 (3.2)		35 (19.3)	11 (17.2)	
Mood disorders	246 (41.0)	78 (35.6)		129 (30.8)	40 (25.8)		117 (64.6)	38 (59.4)	
Personality disorders	60 (10.0)	21 (9.6)		42 (10.0)	13 (8.4)		18 (9.9)	9 (12.5)	
**Head of department n (%)**	136 (22.7)	47 (21.5)	0.776	120 (28.6)	42 (27.1)	0.754	16 (8.8)	5 (7.8)	1
**Working alone n (%)**	144 (24.0)	59 (26.9)	0.411	43 (10.3)	21 (13.5)	0.296	101 (55.8)	38 (59.4)	0.661

### Univariate Analysis

#### Whole Sample

##### Personal Dimension

**Figure 1:**
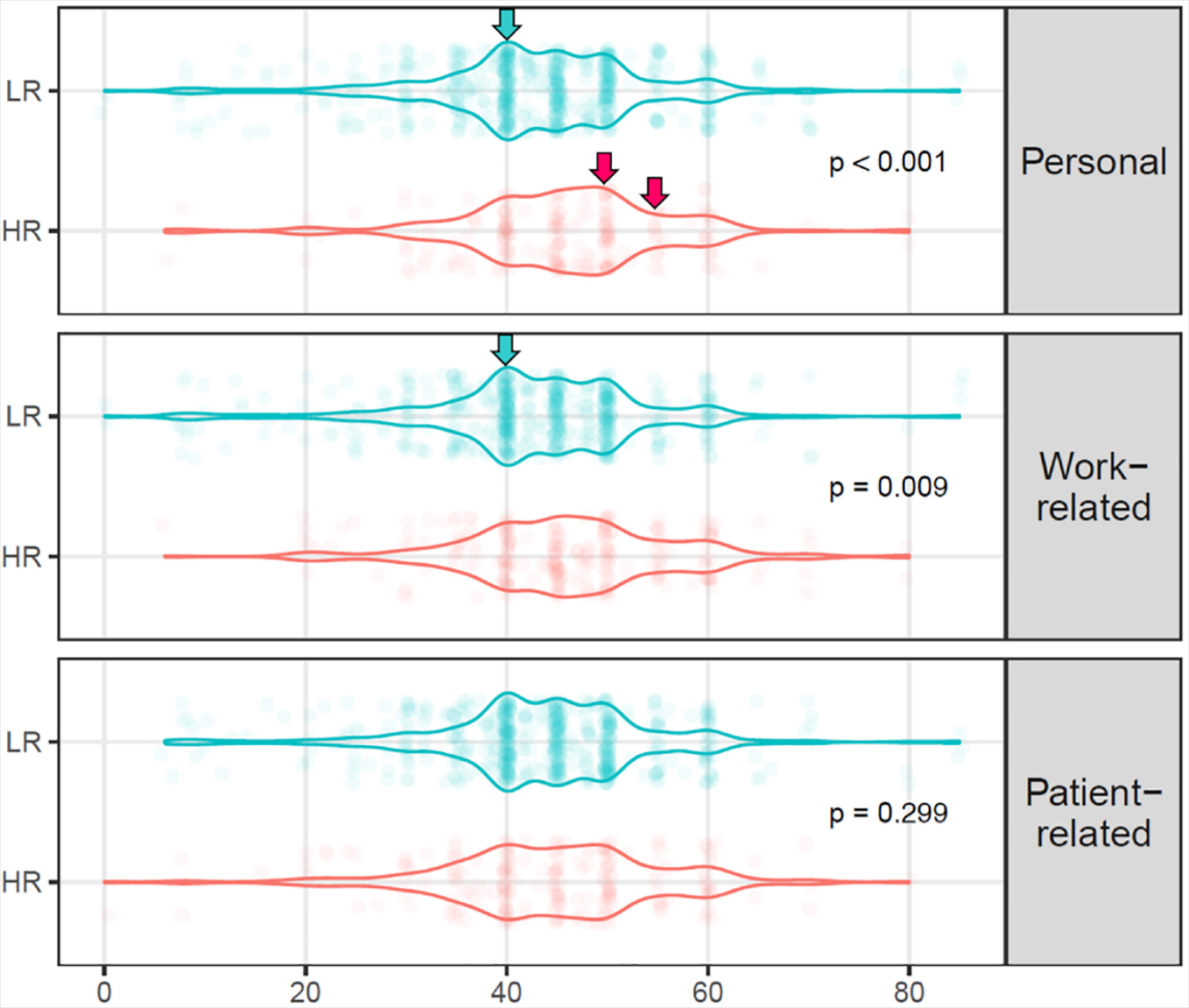
Distribution of the number of hours worked per week by participants in the HR and LR groups for the personal and work- and patient-related dimensions of burnout. Arrows indicate the differences in distribution.

There were more female than male psychiatrists in the HR group for the personal dimension of the CBI (66.0 *vs.* 56.5%, p = 0.016). Mean age was lower for HR than for LR (43.6 *vs.* 46.1 years; p = 0.016). Private practice was associated with a lower risk of burnout on this dimension (17.0 *vs.* 29.3% for public practice, p < 0.001), prompting us to stratify the sample into public- *versus* private-sector practitioners. There was higher proportion of more experienced physicians in the LR group than in the HR group. Unsurprisingly, a good atmosphere at work was less frequent in the HR group (61.5 *vs.* 83.4%, p < 0.001). The number of hours worked per week was higher for the HR group (45.5 *vs.* 43.1 h, p = 0.001) (**Figure 1**). Among all treated disorders and compared to LR, the proportion of participants who mainly treated patients with mood disorders was lower in the HR group (30.0 *vs.* 40.9%, p = 0.004), whereas the proportion of participants who mainly treated schizophrenia/psychosis was higher in this group (41.9 *vs.* 31.6%, p = 0.008).

##### Work-Related Dimension

Women did not significantly predominate in the HR group when the work-related dimension was considered. Again, psychiatrists working in the private sector were significantly less prone to meet the criteria for a higher risk of burnout (23.8 *vs.* 32.5%, p = 0.013).The number of hours worked per week followed the same pattern as that described above for the personal dimension. Proportions of mood disorders and schizophrenia/psychosis as mainly treated disorders were again lower (29.7 *vs.* 43.4%, p = 0.0002) and higher (41.8 *vs.* 29.0%, p = 0.0004) in the HR group, respectively.

##### Patient-Related Dimension

Interestingly, psychiatrists did not describe the doctor-patient relationship as being associated with an increased risk of burnout, whichever associated variable or subsample was examined.

#### Psychiatrists Working in the Public Sector

A total of 587 psychiatrists defined themselves as working either exclusively or mainly in the public sector.

##### Personal Dimension

The proportion of female psychiatrists was higher in the HR group, though this difference was not significant (69.8 *vs.* 61.2%, p = 0.059). Neither age nor years after graduation was associated with a risk of personal burnout. The number of hours worked per week was higher in the HR group (45.1 *vs.* 42.4 h, p < 0.001), while good atmosphere at work was less frequent (60.2 *vs.* 80.2% p < 0.001). Though psychiatrists working four or more night shifts per month were more frequent in the HR group, this difference was not significant (15.4 *vs.* 22.1%, p = 0.056).

##### Work-Related Dimension

The proportion of female psychiatrists was higher in the HR group, though this difference was not significant (68.8 *vs.* 60.5%, p = 0.054). The number of hours worked per week and number of consultations per week were significantly higher in the HR group (44.9 *vs.* 42.3 h, p = 0.009 and 37.5 *vs.* 32.8 consultations, p = 0.035). Schizophrenia/psychosis as mainly treated disorders was again more frequent in the HR group (52.4 *vs.* 41.6%, p = 0.016).

#### Psychiatrists Working in the Private Sector

A total of 253 psychiatrists defined themselves as working either exclusively or mainly in the private sector. As mentioned earlier, most of them were in the LR group. The number of hours worked per week was higher in the HR group (44.9 *vs.* 42.3 h, p < 0.009) in this subsample.

### Multivariate Analysis

A multivariate analysis including all variables revealed that a poor atmosphere at work (OR: 2.88, 95% CI [1.97, 4.20]), more hours worked per week (OR: 1.23 [1.04, 1.47]), and four or more night shifts per month (OR: 1.63 [1.06, 2.48]) were independently associated with HR on the global CBI scale.

### Contextual Questions

We also asked questions enabling us to appraise more qualitatively the perceived conditions of the psychiatrists’ working environment. In particular, they allowed us to pinpoint the nature of the stress associated with work, disentangling stress generated by the workplace climate from stress caused by actual clinical practice. We also distinguished between potential external occupational stressors brought about by new challenges and by changes in society and expectations of care and possible internal ones arising from changes within the psychiatric culture, such as the growing predominance of a neuroscience-based approach to care or the imposition of guidelines.

**Figure 2:**
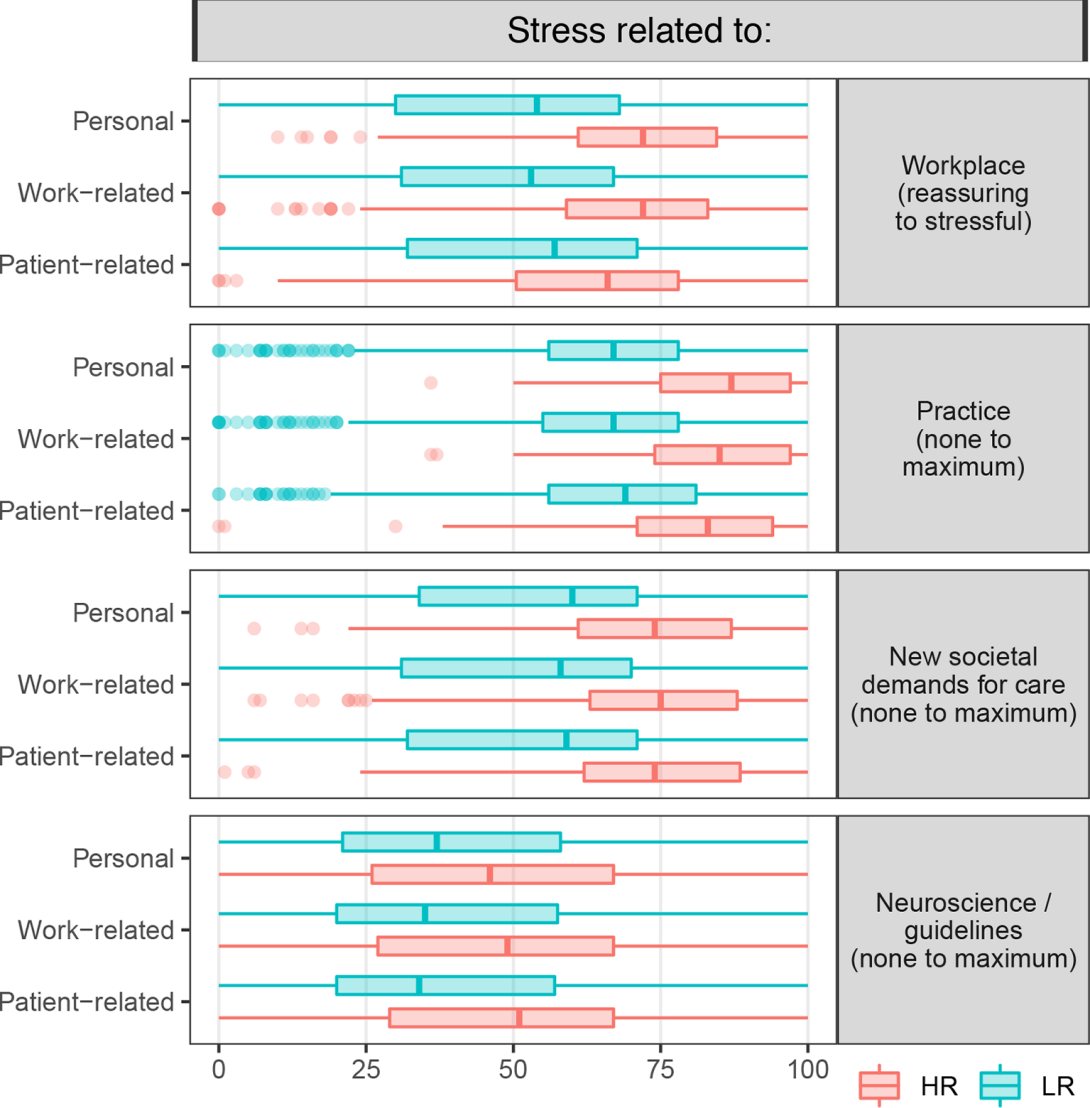
Boxplot distribution of individual responses to the four additional questions appraising the working environment. The difference in the distribution of ratings on the visual analogue scale between the LR and HR groups was highly significant (p < 0.001) for each question.

**Figure 2** shows that stress caused by the working environment and stress generated by clinical practice were both highly significantly associated with a high risk of burnout across all groups (total sample, public- and private-sector subsamples) and dimensions. Social pressure and expectations of care, together with new challenges in psychiatric practice, were also seen as significant stressors by participants in the HR group. By contrast, the neuroscience-based approach and imposition of guidelines distinguished less clearly between the LR and HR groups, with nonsignificant mean differences for the personal dimension in the private-sector subsample.

### Additional Comments

Participants were also invited to freely express themselves about any topic they chose at the end of the questionnaire. In both the LR and HR groups, practitioners stated that, far from being wearisome, taking care of patients was often rewarding. They also acknowledged the deterioration in working conditions, with the burden of clerical work increasing at the cost of patient care. In contrast to the LR group, practitioners in the HR group indicated that they had taken (or were planning to do so in the near future) important decisions about their working status. Several psychiatrists, all in the HR group, stated that they had (or were about to) resigned from their position of public-sector practitioner/head of department or planned to take early retirement. They often described what had triggered this decision (*e.g.* “It was after a week of total insomnia related to the sudden accumulation of administrative demands: bed closures, merging of departments, and certification”). Most of the “recently resigned from the public sector” or “early-retirement prone” individuals stated that their decision had been taken “despite several years voluntarily shouldering major institutional responsibilities” and having “regretfully noted the lack of respect for, and usefulness of this additional work”, along with “ceaseless reorganizations”.

## Discussion

Results indicated that the 25% of psychiatrists with the highest total CBI scores differed significantly from those with lower CBI scores. In particular, female gender, younger age, practising in the public sector, and working longer hours each week were associated with higher personal burnout scores. Public sector and longer hours were also associated with higher work-related burnout scores. Interestingly, none of the variables we studied, with the exception of a poor atmosphere at work (invariably deleterious), was associated with a higher patient-related burnout score, indicating that the patient-doctor relationship barely contributed at all to burnout in this sample of psychiatrists.

Working longer hours was associated with the highest risk score group of work-related burnout in both public and private sector practitioners, as well as number of consultations per week within the public practice sample. When adjusted on the private practice status, working four or more night shifts per month was significantly associated with a higher risk score of burnout on the personal, work-, and patient-related dimensions. Not surprisingly, a poor atmosphere at work was associated with a higher risk score of burnout. The proportion of private practitioners treating people with schizophrenia was higher in the HR group than in the LR group. Public-sector practitioners in the HR group for both the personal and work-related dimensions treated more patients with schizophrenia than their LR counterparts.

The qualitative measures of potential stressors showed that a nonreassuring working environment and a greater number of stressors while treating patients each were significantly associated with HR. Social pressure and care expectations, together with new challenges to be addressed in psychiatric practice, were also seen as significant stressors. By contrast, paradigm shifts within psychiatry as a discipline were seen as less severe stressors, particularly among the private-sector practitioners.

Interestingly, in the present study, though most of the respondent in the HR group do not reach criteria for a high degree of burn out, the variables associated with this highest risk group overlap with those described in the published literature on burnout among healthcare professionals. Additional precipitating—or more severe existing—factors could be involved to reach the burnout threshold. Here, protected factors are also possibly involved. Although gender has not always been identified as an independent predictor of burnout, some studies have found that female physicians have an increased risk of fatigue, compared with men (24). This finding should be set against data showing that the suicide rate among female physicians is 2.27 times higher than that of women in the general population. It is 1.41 times higher for male physicians (25). Frequent call duties and long working hours have also been found to contribute to burnout (26).

A recent systematic review and meta-analysis exploring burnout among French physicians yielded an estimated prevalence of 49% for severe burnout, with higher levels among emergency practitioners and junior (27). These data are in line with a recent meta-analysis estimating the prevalence of burnout to be 44.2% (33.4–55.0%) among medical students before residency, questioning the assumption that residency plays an intrinsic and deleterious role in generating burnout (28). A group of psychiatry residents from 35 French medical faculties was recently compared with residents in other specialities (29). Comparisons revealed that the psychiatry residents had higher rates of drug use disorders and mental health issues. A national survey among Canadian psychiatry residents also found that one-fifth of respondents had high MBI scores for unhealthy coping strategies (30). The present results are in line with the literature showing that younger physicians run nearly twice the risk of burnout as their older colleagues, and onset may occur as early as residency training (31). Physicians working in outpatient settings are also described as experiencing higher burnout than those working in inpatient facilities (32). Some results were conceptually worrying, even if they did not reach significance. These concerned the excessive numbers of residents and heads of department in the HR group (**Tables 1** and **2**).

## General Considerations and Conclusion

Physician burnout has reached epidemic levels all over the world. Whereas burnout among the general working population of the US remained stable at around 28% between 2011 and 2014 (16, 33), the percentage of physicians reporting burnout rose from 45.5 to 54.4% during this period. Burnout rates are thus twice as high in medicine as in other fields. A recent study in China revealed that psychiatrists have a low level of satisfaction, with up to 20% of the studied population reporting that they intended to quit their jobs (34). Another study that also used the CBI questionnaire to examine burnout incidence, this time among psychiatrists in India, revealed that approximately 32% met the criteria for burnout using the total CBI score (35).

The rapid and worldwide spread of burnout among health practitioners evidenced by epidemiological studies and spontaneously perceived by disillusioned physicians is preoccupying and has a cost. At a personal level, burnout is associated with somatic and mental health comorbidities, particularly depression, suicide, and medication and substance abuse. Burnout also threatens the healthcare system, including patient safety, quality of care, and healthcare costs, as it leads to increased MD turnover, early retirement, less than fulltime work, and poor-quality care. High levels of physician burnout can thus be seen as an indicator of poor performance by the underlying system and environment (36). A blog published in September 2018 entitled “Physician Burnout Is A Public Health Crisis: A Message To Our Fellow Health Care CEOs” in the context of the CHCMS Physician Well-Being initiative provided compelling data on the extent of physician burnout and the consequences for healthcare delivery systems in the US. The authors unanimously concluded that physician burnout is a pressing issue of national importance both for these systems and for patients (37).

A recent systematic review and meta-analysis of the studies addressing this issue indicated that intervening to reduce burnout can be effective (38). Interesting prevention and treatment programs aimed at physicians have been developed in several institutions in the US and Europe (39). While most programs developed to reduce burnout in physicians focus on resilience and wellbeing, burnout is also—if not mainly—driven in the medical setting by stressful stimuli. Academic institutions in France could replicate the successful initiatives undertaken by the American College of Physicians, which recently published a position statement, entitled “Putting Patients First by Reducing Administrative Tasks in Health Care” (40). The National Academy of Medicine of the United States has also produced a discussion paper calling for increased research on how changes in physicians’ organizational and practice environments are related to increasing rates of burnout (24). In this context, a recent editorial in *The Lancet* called for “enlightened leaders who recognize that medicine is a human endeavor and not an assembly line” (41).

The present study, designed to pinpoint the factors associated with higher burnout scores on the personal, work-, and patient-related dimensions within a substantial sample of French psychiatrists, yielded some interesting results. To our knowledge, it is the first to have examined factors for perceived work-related burnout among both public- and private-sector psychiatrists in France. However, because it was based on a nonrepresentative population, it had several limitations. In particular, the precise incidence of burnout among French psychiatrists cannot be extrapolated from the present data owing to a selection bias relating to the fact that participants were conference attendees. HR and LR populations were defined here solely for comparison purposes. Results for the HR group should not be unduly generalized as most of the CBI scores of individuals in this group were not high enough for them to have a high degree of burnout. Nevertheless, the present study opens up important avenues for future research on the issue of psychiatrist burnout in the French healthcare system.

There is a growing awareness worldwide of the alarming rate of burnout among practitioners, threatening the survival of many healthcare organizations. Programs need to be developed to reduce burnout in physicians, focusing jointly on increasing resilience and wellbeing, and on changing organizational factors. Several initiatives across the world involving clinicians, and deliberate, sustained, and comprehensive efforts at the highest levels of healthcare organizations will hopefully reverse the rising tide of burnout among clinicians.

## Data Availability Statement

All datasets generated for this study are available upon request.

## Author Contributions

PN, MM, RG, PF and DG contributed to the conception and design of the study. CT organized the database. NL, CT, DG, and PN performed the statistical analysis and interpretation of data for the work. PN and CT wrote the first draft of the manuscript. PN, NL, and CT wrote sections of the manuscript. All authors contributed to manuscript revision, read and approved the submitted version.

## Conflict of Interest

The authors declare that the research was conducted in the absence of any commercial or financial relationships that could be construed as a potential conflict of interest.
